# Can improving working memory prevent academic difficulties? a school based randomised controlled trial

**DOI:** 10.1186/1471-2431-11-57

**Published:** 2011-06-20

**Authors:** Gehan Roberts, Jon Quach, Lisa Gold, Peter Anderson, Field Rickards, Fiona Mensah, John Ainley, Susan Gathercole, Melissa Wake

**Affiliations:** 1 Centre for Community Child Health, Royal Children's Hospital, Parkville, Australia; 2 Murdoch Childrens Research Institute, Parkville, Australia; 3 Deakin Health Economics, Deakin University, Australia; 4 Clinical Epidemiology and Biostatistics Unit, Royal Children's Hospital, Parkville, Australia; 5 Department of Paediatrics, University of Melbourne, Parkville, Australia; 6 Melbourne Graduate School of Education, The University of Melbourne, Australia; 7 Australian Council for Educational Research, Melbourne, Australia; 8 MRC Cognition and Brain Sciences Unit, University of Cambridge, UK

## Abstract

**Background:**

Low academic achievement is common and is associated with adverse outcomes such as grade repetition, behavioural disorders and unemployment. The ability to accurately identify these children and intervene before they experience academic failure would be a major advance over the current 'wait to fail' model. Recent research suggests that a possible modifiable factor for low academic achievement is working memory, the ability to temporarily store and manipulate information in a 'mental workspace'. Children with working memory difficulties are at high risk of academic failure. It has recently been demonstrated that working memory can be improved with adaptive training tasks that encourage improvements in working memory capacity. Our trial will determine whether the intervention is efficacious as a selective prevention strategy for young children at risk of academic difficulties and is cost-effective.

**Methods/Design:**

This randomised controlled trial aims to recruit 440 children with low working memory after a school-based screening of 2880 children in Grade one. We will approach caregivers of all children from 48 participating primary schools in metropolitan Melbourne for consent. Children with low working memory will be randomised to usual care or the intervention. The intervention will consist of 25 computerised working memory training sessions, which take approximately 35 minutes each to complete. Follow-up of children will be conducted at 6, 12 and 24 months post-randomisation through child face-to-face assessment, parent and teacher surveys and data from government authorities. The primary outcome is academic achievement at 12 and 24 months, and other outcomes include child behaviour, attention, health-related quality of life, working memory, and health and educational service utilisation.

**Discussion:**

A successful start to formal learning in school sets the stage for future academic, psychological and economic well-being. If this preventive intervention can be shown to be efficacious, then we will have the potential to prevent academic underachievement in large numbers of at-risk children, to offer a ready-to-use intervention to the Australian school system and to build international research partnerships along the health-education interface, in order to carry our further studies of effectiveness and generalisability.

## Background

Low academic achievement, such as poor literacy, is a common and serious problem, and affects between 10-20% of the population [1,2]. The adverse social and economic long-term outcomes of these difficulties are clear. They include grade repetition, behavioural disorders, mood and self-esteem difficulties and school failure during the school years, [3-5] and unemployment and poverty in adulthood [6].

Learning during childhood is a transactional process between the child and their environment [7]. A poor reader is less likely to read for pleasure and more likely to avoid practice, so that the gap with peers gradually widens until the child starts to fail in school. By the time academic difficulties are evident, which is often not before Grade 3,[1,8] they may already be entrenched. For example, in the Connecticut Longitudinal Study, 70% of children with reading disabilities in 3^rd ^Grade still struggled in 12^th ^Grade [9].

Societies address health and developmental problems using a range of strategies, from the least intensive and most generic (universal prevention) through to the most costly, complex and limited (long-term care for end-stage conditions). From the population perspective, effective prevention is the optimal approach for reasons of both cost and benefit,[10] although evidence as to optimal timing is often meagre [11]. In turn, common problems that develop slowly and thus pose identification challenges - like academic underachievement - may need graded prevention approaches. Thus Mrazek & Haggerty propose that population prevention should range from universal (delivered to whole populations) through selective (population sub-groups at high risk) to indicated (smaller groups with early signs of problems, not yet meeting diagnostic criteria) [12]. As problems crystallise, approaches then move to the individual by case finding, early intervention, treatment and, finally, end-stage care.

Unfortunately, this spectrum of prevention is not yet optimised for academic difficulties. In Australia, universal prevention is offered throughout the preschool years, for example early-life social initiatives to minimise inequalities, promoting shared book-reading with toddlers, and a universal preschool year. In school, children who are identified with early academic difficulties may receive indicated prevention strategies, for example, programs such as Reading Recovery. However, little progress has been made with selective prevention - the crucial intermediate stage when help could be targeted to very young school children at high risk of academic underachievement but who have not yet fallen behind. Systematically delivering a brief, semi-tailored selective prevention intervention to school entry children at risk of academic failure would be a major advance, but, as yet, clear targets for intervention have not been identified.

Working memory has recently been identified as a cognitive process that is vital for learning and may be causal in academic underachievement and learning difficulties, as well as a range of other problems [13]. Working memory is strongly associated with literacy and numeracy skills,[14] and children with poor working memory at school entry are unlikely to reach expected levels of attainment in literacy, maths and science three years later [15]. In population studies, > 80% of primary school children with working memory difficulties on screening (scores < 15^th ^percentile for age) failed to achieve expected levels of achievement in reading and/or maths [13]. Over 90% of 6-11 year-old children with reading difficulties have low working memory skills [16].

Working memory refers to the ability to temporarily store and manipulate information in a 'mental workspace'. Current theory, based on functional activation and brain lesion studies,[13] describes working memory as a multi-component, limited-capacity network linking different cortical centres. It comprises verbal and visuo-spatial short-term memory and a 'central executive' involved in higher level mental processes, attention and executive function [13]. Children with working memory difficulties often make poor academic progress because they become overloaded by classroom demands: they forget crucial task information, fail to follow instructions, and do not complete activities. Learning is thus seriously impeded [13]. Overcoming working memory overload, either by enhancing capacity or by reducing demands, could therefore boost learning. The strong predictive relation between working memory and learning typically persists even after IQ is taken into account,[17] indicating that working memory is more than a mere proxy for intelligence.

Until recently, working memory was considered highly heritable and fixed [18,19]. However, it is now known that it can improve with adaptive training tasks that encourage individuals to work continuously at their personal working memory capacity [20]. This concept has recently been developed into a game-style computerised training program suitable for children as young as 5 years of age by Klingberg and colleagues [20]. Following this program, children with ADHD generalised their new skills and sustained the treatment effect [20]. Functional imaging showed increased activation in the frontal and parietal areas of the brain that are strongly implicated in working memory [21]. A non-randomised trial of 8-11 year-old children in six schools in north-east England reported that this adaptive training can improve both working memory and academic outcomes in the short term [22]. Intervention children also improved in mathematical reasoning by six months (effect size 0.5 SD, p = 0.01), indicating that better working memory may translate directly into more effective learning [22]. IQ scores changed very little. Nor did literacy scores, suggesting that reading problems that are present at age 8-11 years may need more specific and individualised remediation.

Working memory, therefore, now appears to be a strong candidate for a selective prevention intervention for young children at risk of academic underachievement. We now propose to determine whether these benefits translate to younger children screened in the Australian school setting- the next step in determining the true prevention potential of this promising intervention.

## Aims and hypothesis

We aim to trial a targeted approach to prevent poor academic achievement in a selective sample of Grade 1 children identified by screening as having low working memory.

We pose two specific researchable questions in this high-risk group:

1. Can a school-based computerised working memory program have a sustained impact on (a) literacy and numeracy and (b) working memory skills in intervention children, compared with controls who don't receive the program?

2. What are the intervention's costs, compared with its benefits, to children, families and schools?

We **hypothesise **that:

1) Compared with the control group, post randomisation, intervention children will have:

i. higher reading and mathematical scores at 12 (primary outcome) and 24 months,

ii. higher working memory scores at 6 and 12 months, and

iii. better scores on behaviour, attention, social-emotional function and quality of life measures at 12 and 24 months.

2) The intervention will be acceptable and cost-effective to schools and families.

## Methods and design

### Approval and registration

The project is registered with the Australian New Zealand Clinical Trials Registry (ACTRN 12610000486022) and ethics approval was obtained from the Human Research Ethics Committee (HREC 30104) at the Royal Children's Hospital in Melbourne, Australia. Research in schools approval was obtained from the Victorian Department of Education and Early Childhood Development (2010_000800)

### Design

**Figure 1:**
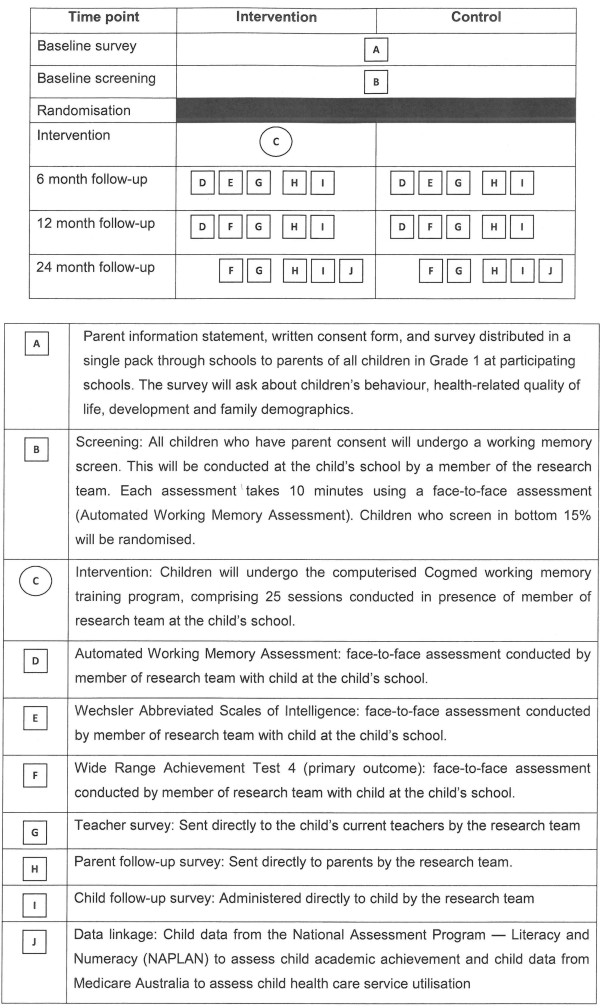
**Graphical representation of trial components**.

The study will be a randomised controlled trial nested in a population-based cross-sectional screening study. Results will be reported according to CONSORT guidelines and the extension report of non-pharmacologic interventions [23,24]. Figure 1 shows the components of the trial graphically.

### Setting

We will approach state primary schools in metropolitan Melbourne (population 4 million in 2009[25]) in the state of Victoria, Australia. There are four school regions (Northern, Eastern, Southern and Western) in Metropolitan Melbourne under the Victorian Department of Education and Early Childhood Development classification. Schools in the Eastern metropolitan region will be approached for this trial. This region serves approximately a quarter of Melbourne's population, servicing around 14,000 students at each year level from diverse socio-economic and cultural backgrounds [26].

### School recruitment

Schools will be randomly selected for invitation to participate in the trial. We will approach each school's principal via telephone for their agreement to take part; we anticipate that about 10-25% of schools will not agree to participate due to time commitment, as per previous studies conducted at the Centre for Community Child Health, Royal Children's Hospital, Melbourne, Australia (the trial's base). If a school does decline, we will go to the next randomly-selected school on our school recruitment list, until we reach the required sample size of 2900 Grade 1 children (Grade 1 refers to the second year of formal primary school education in Victoria, Australia).

Once the school has agreed to participate, we will work with a key liaison person (usually the assistant principal, guidance officer, or junior school coordinator) at each school for the duration of the trial. Before the trial commences, we will meet with all Grade 1 teachers at each school for approximately 30 minutes to describe the expected time commitments, explain the recruitment process, answer any questions they may have and to demonstrate the screening and intervention software.

### Child recruitment

Before recruitment of children commences, we will publicise the trial in the two weeks leading up to recruitment to raise staff and parent awareness of the trial. We will do this through displaying posters on the children's classroom door, including brief segments in the weekly school newsletters and sending home advance-notice postcards to all students in Grade 1 at each participating school.

Recruitment for screening will be staggered over Terms 1 and 2 (February to June in Australia) of the 2012 school year. This will allow screening and intervention to occur in smooth succession within schools - an important factor for success and sustainability.

We will send a trial recruitment pack to the family of each child in Grade 1 via their teacher. This pack will contain a stamped sealable envelope, trial information, consent form, and a brief written parent questionnaire. The questionnaire will collect sociodemographic details, information on potential confounders, and child attributes that may be sensitive to improved learning (e.g. mental health, social skills, and health-related quality of life). It will be written at a Grade 6-7 reading level, with assistance available by phone for parents if needed.

We will seek simultaneous consent for the screen and, in the event of low working memory, the trial. This method minimises two potent sources of bias: (1) control children need not be identified to teachers, and (2) it supports superior intention-to-treat analyses, as all eligible children are included at outcome. In addition, we will seek consent to access Year 3 National Assessment Plan for Literacy and Numeracy (NAPLAN) results (as a further academic outcome) and Medicare health and pharmaceutical utilisation (for the cost-effectiveness analysis) for the trial period.

Parents will be asked to return the completed consent forms and survey in the envelope provided to the child's classroom teacher. A secure box will be supplied to each classroom in which to place the returned envelopes. A reminder pack with the same contents as the original recruitment pack will be sent home with each child if a consent form and survey have not been returned within two weeks. Parents will be asked to return the completed forms within a week if they wish to participate in the trial. A member of the research team will collect the completed surveys and forms from the schools.

### Child screening

All children in Grade 1 who have a completed consent form will be screened for working memory difficulties within two weeks of completing the forms. With the staggered approach, working memory will be screened in Term 1 or 2 of the 4-term year by research assistants at participating schools during school hours. Each research assistant will screen one child at a time, with each screen taking around 10 minutes. A typical school of approximately 60 Grade 1 children would thus be screened in 3 person-days. Up to three research assistants will be available to screen children at each school to minimise disruption to the school.

### Inclusion and exclusion criteria

#### Inclusion

Children with low working memory are defined as those scoring < 25^th ^percentile on both the backward digit recall and the spatial span tasks from the Automated Working Memory Assessment (AWMA), which equates to about 20% of the population [27]. Children in this category will be eligible for the intervention trial.

#### Exclusion

Children with severe disabilities (e.g. cerebral palsy, vision/hearing impairments or pervasive developmental disorders) that do not allow participation in the intervention program will be excluded from the screening and intervention trial. We will screen for these conditions on the initial parent survey and via discussions with the school. Children and families from non-English speaking backgrounds whose English language abilities do not allow them to participate in the intervention, assessments or completion of questionnaires will also be excluded. Although this will affect the generalisability of our results to such children, the aim of the trial is to establish efficacy. Once efficacy has been established, issues of generalisability will be addressed in future research.

### Randomisation

Eligible children will be individually randomised into the 'usual teaching' (control) or 'working memory' (intervention) group, stratified by school. Contamination will be unlikely, as control children are not identified to teachers nor can they access the training program. The randomisation will be conducted by a researcher independent of the research team. Allocation will be concealed from members of the research team involved in outcome assessments for the duration of the trial. The research team will notify parents by mail of their children's results, including group allocation and the remaining steps of the trial for the children with low working memory.

### Intervention delivery training

We will train our staff according to the Cogmed working memory training model of 'coaches' and 'training aides' [28]. The trial's project manager (JQ) was trained in July 2010 as a Cogmed 'coach' by receiving a full day of training from an authorised training provider and delivering the intervention over 5 weeks to 5 children. As a certified Cogmed 'coach',[28] he is now qualified to train the other research assistants to conduct the intervention as 'training aides' in two half-day training sessions. In addition, the Cogmed coach will hold fortnightly meetings with the training aides to discuss and review the intervention's progress and to discuss any difficulties which may arise.

### Intervention

Intervention children will start their adaptive working memory program within six weeks of screening. The intervention trains working memory skills using an interactive and motivating, game-format, computerised training program [20]. It runs for 35 minutes a day for up to 25 sessions over five weeks. All training is conducted at school in small groups of up to four students under supervision of a research assistant. Eight tasks are completed every day. The children train on the same tasks for the first five days. A new task replaces one of the existing tasks on day 6 and every 5^th ^day after this. Within each task, the adaptive nature of the program matches difficulty to the child's current working memory skill on a day-by-day basis, with all tasks increasing in complexity according to the child's current skill.

Each task involves the temporary storage and manipulation of visual-spatial information in a computer game-based format, such as recalling a sequence of animals that light up in a certain order. Motivational features include positive verbal feedback, displaying 'high scores' and accumulation of stars when tasks are successfully completed. A fish tank is displayed when the day's session is completed and award objects, such as shipwrecks, goldfish and turtles, are added each day.

As the schools are geographically close, one researcher will be able to deliver the intervention in up to three schools per day. Control children will not be identified to their teacher and will receive the usual curriculum.

### Measures and procedures

The primary outcome measure for the trial is academic achievement in the intervention group compared with the control group, measured using the *Wide Range Achievement Test (WRAT 4) *[29]. Other outcome measures include working memory, quality of life, social-emotional functioning and health care utilisation. Intelligence quotient will also be screened in the 2 groups. Table 1 summarises timing of outcome measures for the trial and at which time point they will be used.

**Table 1:** Key trial measures

Domain	Measure	T1	T2	T3	T4
Working memory (population screen)	Children with low working memory will be defined as those scoring < 25^th ^percentile on both the backward digit recall and the spatial span tasks from the Automated Working Memory Assessment (AWMA) [27].	•			

Achievement (primary outcome)	*Wide Range Achievement Test (WRAT 4) *[29] is a validated measure of child academic achievement. It yields standard (mean 100, SD 15) reading composite (word reading and sentence comprehension subtests) and maths computation scores. The WRAT will determine if early working memory benefits translate into subsequent learning. Gains at 12 months not sustained at 24 months would indicate that repeated bursts of working memory training may be helpful.			•	•

Working memory	*Automated Working Memory Assessment (AWMA) *is standardised for ages 4-22 years, the AWMA is a PC-based, valid and reliable working memory assessment tool that yields composite and subtest scores (mean 100, SD 15) [27]. We will administer the following subtests: digit recall, listening recall, dot matrix, spatial span and backward digit recall (assessing verbal, visuo-spatial and central executive components of working memory). This will show whether short-term working memory gains are made and sustained over time.		•	•	

Intelligence Quotient	*Wechsler Abbreviated Scales of Intelligence (WASI) *[35] is a brief measure of intellectual ability is standardised for ages 6 to 89. Its 4 subscales yield verbal, non-verbal and composite scores (mean 100, SD 15) that correlate strongly with full scale WISC-III scores, and will allow us to explore differential benefits of the program by underlying cognition.		•		

Health-related quality of life	*Peds-QL™4.0 *[36]. This 23-item measure for 2-18 year olds provides Total, Physical and Psychosocial scores and is widely used as a proxy for child health-related quality of life.	•	•	•	•
	
	The *PedsQL - SF15*, is a15-item validated child self-report measure for children aged 5 to 7 years yielding a score with a possible range 0-100 [37].			•	•

Quality adjusted life years	*Child Health Utility 9D (CHU-*9D)[38] is a self-report health-related quality of life questionnaire is validated for children aged 7 to 11, and will be used at the 12 and 24 follow-up to calculate child-reported quality adjusted life years (QALYs) for use in cost-consequences analysis.			•	•

Behaviour	*Strengths and Difficulties Questionnaire Parent Report English (Australian) *[39]. Widely used, well-validated 25-item questionnaire probing behaviour in 4-16 year olds; yields Prosocial and Total Problem scores as well as emotional, conduct, hyperactivity, and peer subscales.	•	•	•	•
	
	*SDQ teacher version*, for a multi-informant perspective on the program's mental health impacts.		•	•	•

Academic	National Assessment Program - Literacy and Numeracy (NAPLAN)[40] is an annually administered test for all students in Australian in Years 3, 5, 7 and 9. The assessment consists of four domains of reading, writing, language conventions (spelling, grammar and punctuation) and numeracy.				•

Health service utilisation	Medicare data will be accessed from Medicare Australia, which is an Australian Government agency delivering a range of payments and services Australian citizens. Medicare enables Australians have access to free or low-cost medical, optometric and hospital care through a universal health service. Medicare tracks data on health service utilisation from public and private health services [41].				•

T1 = Baseline, T2 = 6 months post-randomisation, T3 = 12 months post-randomisation, T4 = 24 months post-randomisation

We will proceed directly from screen to intervention, with baseline assessments that include working memory screening and assessments of quality of life, social-emotional functioning and health care utilisation, for the following reasons:

(i) this minimises time between screening early in the year and the mid-year intervention - allowing children to make useful learning gains during the remainder of the school year.

(ii) a detailed face-to-face baseline assessment would alert and unblind teachers to control children as they would be aware of which children who had low working memory in their classroom, rather than just the children in the intervention group, making contamination more likely.

The working memory screen correlates strongly with the full score, providing a good proxy in multivariable analyses adjusting for baseline.

### Process evaluation

All teachers will complete written surveys at six months post-randomisation documenting their perceptions of program implementation, acceptability, barriers to implementation, and perceived harms and benefits. The researchers implementing the program will use standardised logs to prospectively record time spent, travel costs and other resources used in intervention preparation and delivery, student attendance for each session and any school-specific issues that arise in the delivery of each session (such as IT difficulties).

### Economic evaluation

The economic evaluation of the intervention will be a two-stage analysis. We will use cost-consequences analysis as a first step to compare any incremental costs of the intervention (costs accrued in the intervention arm, from intervention and resource use over the period of follow-up, compared to costs accrued in the control arm) to all primary and secondary outcomes, expressed in their natural units of measurement. We will then proceed to cost-effectiveness analysis to compare incremental costs to difference in the WRAT4, the pre-specified primary outcome of academic achievement [30,31].

All analyses will be conducted from health and education service, as well as the broader societal, perspectives, as interventions cost-effective from a service perspective can add substantially to family costs [32]. Research assistants will prospectively record resources used in screening and intervention delivery. Parents will retrospectively recall service use over the previous year at recruitment, 12 and 24 months. Parental recall of child service, financial and time resource use over periods up to one year can capture family resource use inside and outside the formal health care sector [33]. Measured resource use will be valued using existing unit cost estimates (e.g. education department salary scales, Medical Benefit Schedule fee rates). Uncertainty in cost and outcome data and sensitivity of economic evaluation results to chosen methods of evaluation will be tested by extensive sensitivity analyses [31].

### Measurement training - face to face measures

Measurement training will be conducted by other research staff at the Centre for Community Child Health who have previously used the measure. The training will involve familiarisation with the assessment components, how they are delivered and scoring. Role plays will be conducted to allow for mock assessments to be conducted in the presence of the trainer. Staff will observe the assessments being used either in a clinic or as part of another research project. In addition, the first assessment conducted by each staff member will be observed by a more experienced member of the research team. A fortnightly meeting will be conducted to ensure assessment fidelity and to troubleshoot any issues which may arise from the assessments.

### Sample size

We aim for 175 children in each trial arm, 350 in total, available for primary endpoint of academic achievement score comparison at 12 and 24 months, providing 80% power to detect a clinically important difference of 0.3 SD at a significance level of 0.05. A teacher-related cluster effect is likely to have a negligible effect on power especially by 12 and 24 months, by which time participants will usually have changed teachers.

**Figure 2:**
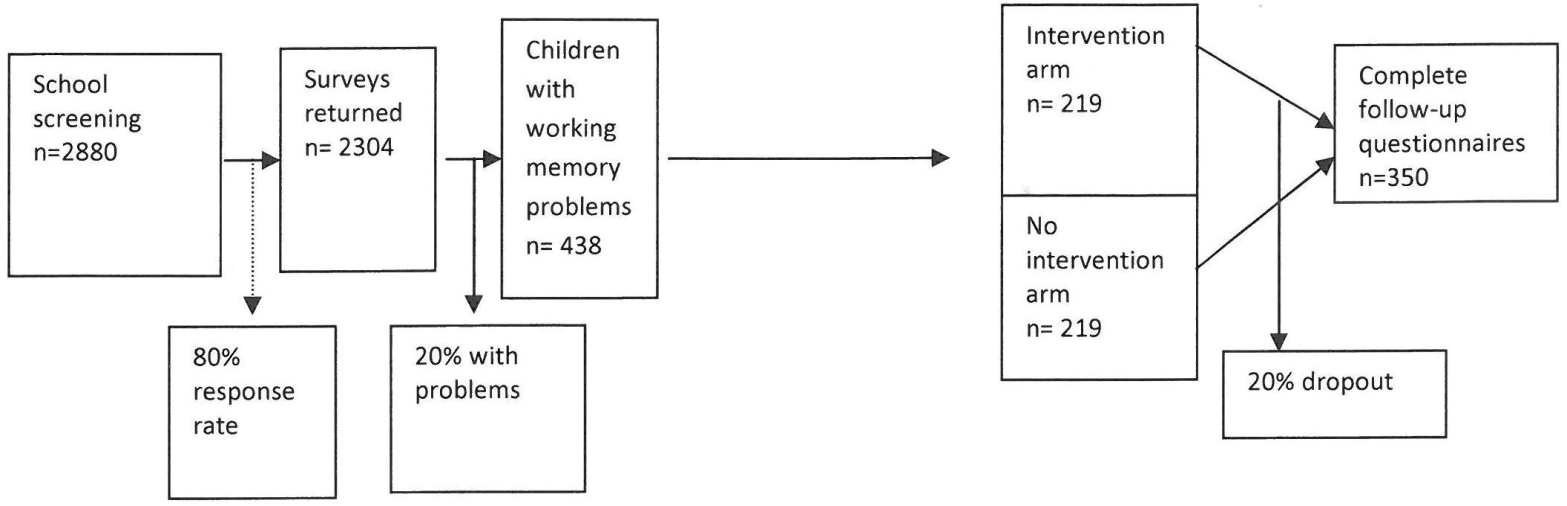
**Flowchart of study participants**.

We will therefore aim to recruit Grade 1 children from 48 schools. Assuming an 'average' government school has 3 Grade 1 classes, each with approximately 20 children, we will aim to approach 2880 children and expect that around 80% will participate in the trial, 2304 in total. Of these children we expect to identify around 440 with working memory difficulties (19%) which, allowing for up to 20% attrition, will give us our final sample size of around 350 (Figure 2).

### Statistical Analysis

For both hypotheses, analyses will be based on the intention-to-treat principle and will compare outcomes (and costs) post-intervention and at 12 and 24 months between the intervention and control arms, using continuous standard scores on the primary (WRAT4) and secondary outcomes. We will present results of both unadjusted analyses and analyses adjusted for potential confounding factors (including age, gender and socio-demographic risk factors). Clustering of children within schools and repeated measures within children will be accounted for using regression techniques that respect these structures [34].

## Discussion

School outcomes largely determine a society's social, health and economic capital. A successful start to formal learning in school is formative to these outcomes. The Australian government recognises this and considers improving literacy and numeracy outcomes of the nation's children a national priority. A targeted prevention approach that could identify and genuinely help at-risk children early in their school career would be a major advance, nationally and internationally. This intervention could systematically address a modifiable problem that is likely to prevent an optimal start to learning in school.

Working memory deficits are now known to be one such modifiable problem that often underlie academic underachievement, and therefore pose a major child health, educational and societal burden. Working memory deficits can be identified early - even before academic difficulties become obvious. Promising new evidence, outlined above, suggests that working memory deficits can be improved by a brief training intervention in the early school years. If we can translate this new evidence to show that this intervention is efficacious at the population level, and that this in turn prevents academic underachievement, then a potent new preventive strategy could become available to many thousands of at-risk children.

Our proposed intervention trial has several strengths. It will:

• embrace a selective prevention strategy by targeting younger children, prior to academic difficulties becoming established,

• assemble a random sample of schools from across the socio-demographic spectrum,

• be randomised and controlled - the strongest possible design for establishing efficacy,

• be fully blinded - thus avoiding an important source of bias in outcomes,

• be considerably larger than previous studies - and thus able to define the potential effects much more precisely,

• report outcomes to 12 and 24 months - establishing long-term effects on learning, and

• incorporate a health economic analysis - informing policy decisions as to the program's value.

The major limitation of this trial is a lack of generalisability to non-English speaking populations, and this needs to be addressed in future effectiveness studies.

Our trial sets out to translate the exciting initial working memory intervention program findings to the Australian population in a large random sample of schools, within a current policy framework, and with analysis of real costs and benefits. No such trial has been previously reported, either in Australia or internationally. If cost-effective, we expect the following outcomes:

• strong evidence that addressing working memory problems can improve academic outcomes.

• a ready-to-use intervention for the Australian school and policy system, which can be replicated internationally.

In summary, this trial has the potential to make an original and significant contribution to providing children with **a successful start to formal learning**, with flow-on effects throughout their schooling and later life.

## Competing interests

All authors declare that GR, JQ, LG, PA, FR, FM, JA, SG, MW, their spouses, partners or children have no financial and non-financial relationships or interests that may be relevant to the submitted work.

## Authors' contributions

GR and MW conceived the study. JQ drafted the current manuscript. GR, MW, JQ, LG, PA, JA, FR, FM and SG have participated in the design of the study and edited the current manuscript. All authors have read and approved the final manuscript.

## Pre-publication history

The pre-publication history for this paper can be accessed here:

http://www.biomedcentral.com/1471-2431/11/57/prepub
